# Supervised machine learning of ultracold atoms with speckle disorder

**DOI:** 10.1038/s41598-019-42125-w

**Published:** 2019-04-04

**Authors:** S. Pilati, P. Pieri

**Affiliations:** 1 School of Science and Technology, Physics Division, Università di Camerino, 62032 Camerino, (MC) Italy; 2 INFN, Sezione di Perugia, 06123 Perugia, (PG) Italy

## Abstract

We analyze how accurately supervised machine learning techniques can predict the lowest energy levels of one-dimensional noninteracting ultracold atoms subject to the correlated disorder due to an optical speckle field. Deep neural networks with different numbers of hidden layers and neurons per layer are trained on large sets of instances of the speckle field, whose energy levels have been preventively determined via a high-order finite difference technique. The Fourier components of the speckle field are used as the feature vector to represent the speckle-field instances. A comprehensive analysis of the details that determine the possible success of supervised machine learning tasks, namely the depth and the width of the neural network, the size of the training set, and the magnitude of the regularization parameter, is presented. It is found that ground state energies of previously unseen instances can be predicted with an essentially negligible error given a computationally feasible number of training instances. First and second excited state energies can be predicted too, albeit with slightly lower accuracy and using more layers of hidden neurons. We also find that a three-layer neural network is remarkably resilient to Gaussian noise added to the training-set data (up to 10% noise level), suggesting that cold-atom quantum simulators could be used to train artificial neural networks.

## Introduction

Machine learning techniques are at the heart of various technologies used in every day life, like e-mail spam filtering, voice recognition software, and web-text analysis tools. They have already acquired relevance also in physics and chemistry research. In these fields, they have been employed for diverse tasks, including: finding energy-density functionals^1–4^, identifying phases and phase transitions in many-body systems^5–11^, predicting properties such as the atomization energy of molecules and crystals from large databases of known compounds^12–14^, or predicting ligand-protein poses and affinities for drug-design research^15–18^. Among the various machine learning methodologies, supervised machine learning has been put forward as a fast, and possibly accurate, technique to predict the energies of quantum systems exploiting the information contained in large datasets obtained using computationally expensive numerical tools^19^.

Computational physicists and chemists have already demonstrated that supervised machine learning can be used, in particular, to determine the potential energy surfaces for molecular dynamics simulations of materials, of chemical compounds, and of biological systems^20–26^. This allows one to avoid on-the-fly quantum mechanical electronic-structure computations, providing a dramatic speed-up that makes larger scale simulations of complex systems as, e.g., liquid and solid water, feasible^27^. However, it is not yet precisely known how accurately the statistical models commonly employed in supervised machine learning can describe quantum systems. In general, the accuracy achievable by these statistical models, chiefly artificial neural networks, depends on various important details, including the depth and the connectivity structure of the neural network, the size of the training set, and the type of regularization employed during the training process to avoid the pervasive over-fitting problem^28^. Also the choice of the features adopted to represent the quantum system of interest plays a crucial role; in fact, substantial research work has been devoted to the development of efficient representations (see, e.g., refs^17,29,30^). It is natural to expect that addressing models that describe highly tunable and easily accessible experimental setups could shed some light on this important issue.

These considerations lead us to focus on ultracold atom experiments. These systems have emerged in recent years as an ideal platform to investigate quantum many-body phenomena^31,32^. They allowed experimentalists to implement archetypal Hubbard-type models of condensed matter physics^33^ and even the realization of programmable simulators of quantum spin Hamiltonians^34^. One can envision the use of these analog quantum simulators as computational engines to create datasets to be fed to supervised machine learning methods, providing data to train artificial neural networks even for models that defeat computational techniques. One example is the fermionic Hubbard model, that has been implemented in various cold-atom laboratories^35–37^. In fact, recent cold-atom quantum simulations of the Hubbard-model have been analyzed via machine learning techniques^38^.

One of the quantum phenomena that received most consideration by cold-atom researchers is the Anderson localization transition in the presence of disorder^39–43^. This phenomenon consists in the spatial localization of the single particle states, determining the absence of transport in macroscopic samples^44^. Unlike conventional condensed matter systems, which inherently include a certain amount of impurities, in cold-atom setups disorder is introduced on purpose. The most frequently used technique consists in creating optical speckle fields by shining lasers through rough semitransparent surfaces, and then focusing them onto the atomic cloud. These speckle fields are characterized by a particular structure of the spatial autocorrelation of the local optical field intensities^45,46^. These correlations have to be accounted for in the modeling of cold-atom experiments with speckle fields^47,48^. Indeed, they determine the position of the mobility edge^49–51^, namely the energy threshold that in three dimensional systems separates the localized states from the extended ergodic states. In low dimensional configurations, any amount of disorder is sufficient to induce Anderson localization. However, the speckle-field correlations determine the transport properties and even the emergence of so-called effective mobility edge, i.e. energy thresholds where the localization length changes abruptly^52–54^.

In this article we perform a supervised machine learning study of the lowest three energy levels of a one-dimensional quantum particle moving in a disordered external field. This model is designed to describe an alkali atom exposed to a one-dimensional optical speckle filed, taking into account the detailed structure of the spatial correlations of the local intensities of the speckle field. This is in fact the setup implemented in the first cold-atom experiments on Anderson localization^39,40^. The first task we address is to determine the energy levels of a large set of speckle-field instances via a high-order finite difference formula. Next, we train a deep artificial neural network to reproduce the energy levels of this training set, and we then employ the trained neural network to predict the energy levels of previously unseen speckle-field instances.

The main goals of this study are (i) to analyze how accurately deep neural networks can predict low-lying energy levels of (previously unseen) system instances, (ii) to quantify how this accuracy depends on the depth and width of the network, (iii) to verify if and how the overfitting problem can be avoided, and how large the training set has to be to achieve this, (iii) to check if and how accurately excited state energies can be predicted, compared to ground-state energy levels. Furthermore, in view of the possible future use of cold-atom quantum simulators to provide data for supervised machine learning tasks, we analyze if and to what extent deep neural networks are resilient with respect to noise present in the training data. Such noise is indeed an unavoidable feature of any experimental outcome.

The main result we obtain is that, given a computationally affordable number of system instances for training, a neural network with three hidden layers can predict ground state energies of new system instances with a mean quadratic error well below 1% (relative to the expected variance); this error appears to systematically decrease with training-set size. Higher energy levels can be predicted too, but the accuracy of these predictions is slightly lower and requires the training of deeper neural networks. We also show that if one has only small or moderately large training sets (including order of 10^3^ instances, as in some previous machine learning studies) the overfitting problem does indeed occur, and it has to be minimized via an appropriate regularization, that we quantify. Another important finding we report here is that a deep neural network (with three layers of hidden neurons) is extremely robust against noise in the training set, providing essentially unaffected predictions for previously unseen instances up to almost 10% noise level in the training data.

## Model

The model we consider is defined by a Hamiltonian operator that in coordinate representation reads:

1 $$\hat{H}=-\,\frac{{\hslash }^{2}}{2m}\frac{{{\rm{d}}}^{2}}{{\rm{d}}{x}^{2}}+{V}_{{\rm{d}}}(x),$$

where *ħ* is the reduced Planck’s constant and *m* the particle mass. *V*_d_(*x*) is a disordered external field, designed to represent the potential energy of an atom subject to an optical speckle field. Experimentally, these optical fields are generated when coherent light passes through, or is reflected by, rough surfaces. In the far field regime, a specific light intensity pattern develops, commonly referred to as optical speckle field. In cold-atom experiments, this optical speckle field is focused onto the atomic cloud using a converging lens.

A numerical algorithm to generate the intensity profile of a speckle field is based on the following expression^55^:

2 $${V}_{{\rm{d}}}(x)={V}_{0}|{F}^{-1}[W(\xi )F[\phi ](\xi )](x{)|}^{2}.$$

Here, the constant *V*_0_ corresponds to the average intensity of the speckle field, while we denote with

3 $$F[\phi ](\xi )=\int \,{\rm{d}}x\phi (x){e}^{-i2\pi \xi x}$$

the Fourier transform of the complex field *φ*(*x*), whose real and imaginary part are independent random variables sampled from a Gaussian distribution with zero mean and unit variance. *F*^−1^ indicates the inverse Fourier transform. The function *W*(*ξ*) is a filter defined as

4 $$W(\xi )=\{\begin{array}{cc}1 & {\rm{if}}\,|\xi |\le w/2\\ 0 & {\rm{if}}\,|\xi | > w/2\end{array},$$

where *w* is the aperture width, which depends on the details of the optical apparatus employed to create and focus the speckle field, namely the laser wavelength, the size (illuminated area) and the focal length of the lens employed for focusing. We consider blue detuned optical fields, for which the constant *V*_0_ introduced in Eq. (2) is positive.

In the numerical implementation, the Gaussian random complex field *φ*(*x*) is defined on a discrete grid: *x*_*g*_ = *gδx*, where *δx* = *L*/*N*_*g*_, *L* is the system size, and the integer *g* = 0, 1, …, *N*_*g*_ − 1. The number of grid points *N*_*g*_ shall be large, as discussed below. The continuous Fourier transform is henceforth replaced by the discrete version. Periodic boundary conditions are adopted, and the definition (2) is consistent with this choice, i.e. *V*_d_(*L*) = *V*_d_(0).

For a large enough systems size *L*, the optical speckle field is self-averaging, meaning that spatial averages coincide with the average of local values over many instances of the speckle field, indicated as 〈*V*_d_(*x*)〉_d_. These instances are realized by choosing different random numbers to define the complex field *φ*(*x*). The probability distribution of the local speckle-field intensity *V*_loc_ = *V*_d_(*x*), for any *x*, is *P*(*V*_loc_) = exp(−*V*_loc_/*V*_0_)/*V*_0_ for ${V}_{{\rm{loc}}}\ge 0$, and *P*(*V*_loc_) = 0 otherwise. It follows that, for large enough *L*, the average speckle-field intensity 〈*V*_d_(*x*)〉_d_ = *V*_0_ is equal to the standard deviation $\sqrt{{\langle {V}_{{\rm{d}}}{(x)}^{2}\rangle }_{{\rm{d}}}-{V}_{0}^{2}}={V}_{0}$. Therefore, *V*_0_ is the unique parameter that determines the amount of disorder in the system.

The local speckle-field intensities at different positions have statistical correlations, characterized by the following spatial autocorrelation function:

5 $${\rm{\Gamma }}(x)=\langle {V}_{{\rm{d}}}(x^{\prime} ){V}_{{\rm{d}}}(x^{\prime} +x)\rangle /{V}_{0}^{2}-1={[\sin (\pi wx)/(\pi wx)]}^{2}.$$

One notices that the inverse of the aperture width *w* determines the correlation length, i.e. the typical size of the speckle grains. We will indicate this length scale as *γ* = *w*^−1^, which corresponds to the first zero of the correlation function Γ(*x*). The correlation length allows one to define an energy scale, dubbed correlation energy, defined as *E*_c_ = *ħ*^2^/(2*mγ*^2^).

In the following we consider the system size *L* = 20*γ*, with a number of grid points *N*_g_ = 1024. Notice that with this choice one has $\delta x\ll \gamma$, so that the discretization effect is irrelevant. Furthermore, the speckle-field intensity is fixed at the moderately large value *V*_0_ = 5*E*_c_. We point out that we choose to normalize the optical speckle field so that its spatial average over the finite system size *L* exactly corresponds to *V*_0_, for each individual instance, thus eliminating small fluctuations due to finite size effects.

**Figure 1:**
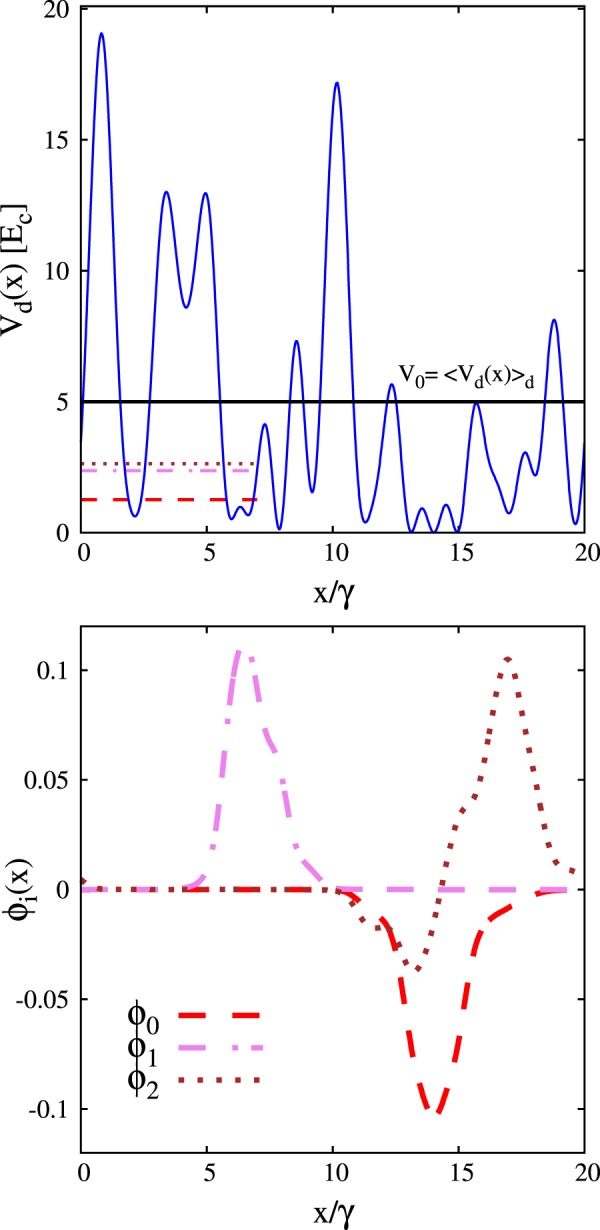
Upper panel: local intensity *V*_d_(*x*) of a typical instance of optical speckle field, as a function of the spatial coordinate *x*/*γ*. The energy unit is the correlation energy *E*_c_ = *ħ*^2^/(2*mγ*^2^), defined by the spatial correlation length of the speckle field *γ*. The continuous horizontal (black) line indicates the average over many speckle-field instances of the optical speckle field intensity *V*_0_ = 〈*V*_*d*_(*x*)〉_*d*_. Lower panel: profile of the three lowest-energy wave functions of the speckle field instance displayed in the upper panel. The corresponding energy levels are indicated by the three horizontal segments in the upper panel.

The local intensity profile of a typical instance of optical speckle field is displayed in the upper panel of Fig. 1. The continuous horizontal line indicates the average intensity *V*_0_. The lower panel displays the three eigenfunctions *ϕ*_*i*_(*x*), with *i* = 0, 1, 2, corresponding to the lowest energy levels. They solve the Schrödinger equation $\hat{H}{\phi }_{i}(x)={e}_{i}{\phi }_{i}(x)$ with eigenvalues *e*_*i*_. These energy levels are indicated by the three horizontal segments in the upper panel of Fig. 1. The wave functions and the corresponding energy levels are computed via a finite difference approach, employing the grid points *x*_*g*_ defined above, using a highly accurate 11-point finite difference formula. This makes the discretization error negligible. One notices that the wave functions *ϕ*_*i*_(*x*) have non-negligible values only in a small region of space. This is consistent with the Anderson localization phenomenon, which in one-dimensional configurations is expected to occur for any amount of disorder, as predicted by the scaling theory of Anderson localization^56^. Indeed, one might also notice that the node of the wave function corresponding to the first excited state is in a region of vanishing amplitude and is therefore barely visible.

**Figure 2:**
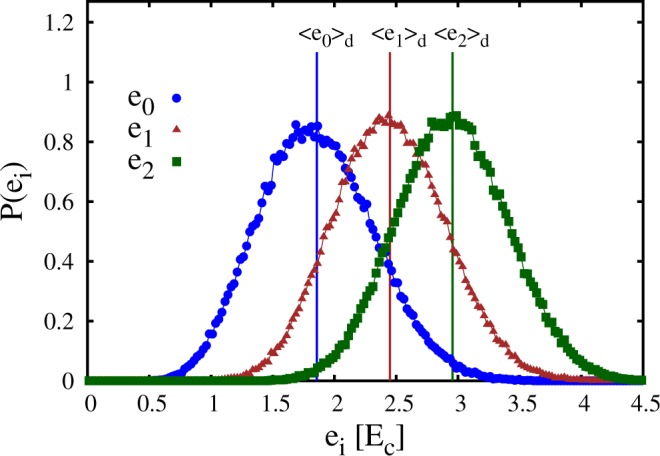
Probability distribution *P*(*e*_*i*_) of the first three energy levels *e*_0_, *e*_1_, and *e*_2_. The vertical segments indicate the corresponding averages over many speckle-field instances 〈*e*_0_〉_d_, 〈*e*_1_〉_d_, and 〈*e*_2_〉_d_. The energy unit is the correlation energy *E*_c_.

Clearly, the energy levels *e*_*i*_ randomly fluctuate for different instances of the speckle field. Their probability distribution is shown in Fig. 2, where the averages over many speckle-field instances 〈*e*_*i*_〉_d_ are also indicated with vertical segments. One notices that the probability distribution of the ground-state energy *e*_0_ is slightly asymmetric, while the distributions of the excited energy levels *e*_1_ and *e*_2_ appear to be essentially symmetric. Other properties of quantum particles in an optical speckle field, such as the density of states, have been investigated in refs^47,57^.

## Methods

The first step in a supervised machine learning study consists in choosing how to represent the system instances. One has to choose *N*_*f*_ real values that describe the system, all together constituting the so-called features vector. One natural choice would consist in choosing the speckle field values *V*_d_(*x*_*g*_) on the *N*_*g*_ points of the spatial grid defined in Sec. 1. Indeed, if the grid is fine enough these values fully define the system Hamiltonian. However, since *N*_*g*_ has to be large, this choice leads to a pretty large features vector, making the training of a deep neural network with many neurons and many layers rather computational expensive. This approach was in fact adopted in a recent related article^19^. The problem of the large feature vector was circumvented by employing so-called convolutional neural networks. In such networks the connectivity structure is limited. This reduces the number of parameters to be optimized, making the training more computationally affordable. The connectivity structure is in fact designed so that the network can recognize the spatial structures in the feature vector, somehow automatically extracting the relevant details from a large feature space.

**Figure 3:**
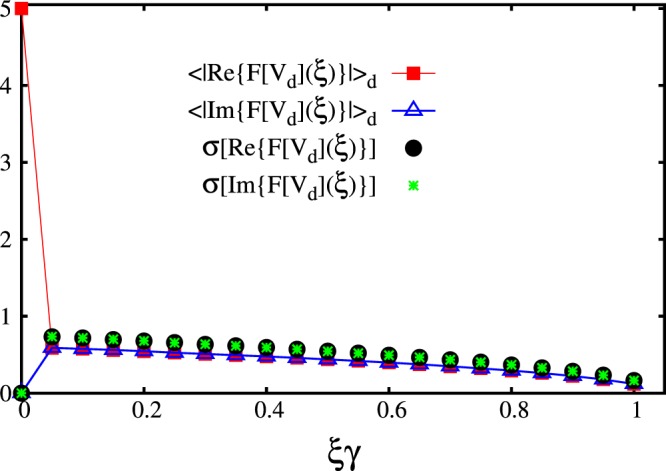
Average over many speckle-field instances of the absolute value of the Fourier components of the speckle field. The (red) squares correspond to the real part. The (blue) open triangles correspond to the imaginary part. The (black) circles and the (green) stars indicate the standard deviations of the real part and of the imaginary part, respectively.

In this article we adopt a different strategy. The definition of the optical speckle field in Eq. (2) and the structure of the spatial correlations described in Sec. 1 suggest that one can construct a more compact system representation by switching to the Fourier space. In fact, it is easy to show that the (discrete) Fourier transform of the speckle field *F*[*V*_d_](*ξ*) has a finite support, limited to the interval *ξ*∈[−*w*:*w*]. This limits the number of nonzero Fourier components. Since the Fourier grid spacing is *δξ* = 1/*L*, one expects to have 42 nonzero (complex) Fourier components for our choice of system size *L* = 20*γ*. One should also consider that the Fourier transform of a real signal has the symmetry *F*[*V*_d_](−*ξ*) = *F*[*V*_d_](*ξ*)^*^. This further limits the number of nonzero independent variables, leaving us with a feature vector with only *N*_*f*_ = 42 (real) components. In Fig. 3 we plot the average over many speckle-field instances of the absolute value of the real and imaginary parts of the Fourier components *F*[*V*_d_](*ξ*). Only the positive semiaxis $\xi \ge 0$ is considered, due to the symmetry mentioned above. It should also be pointed out that due to the choice of normalization discussed in Sec. 1, the real part of the Fourier transform at *ξ* = 0 is fixed at Re{*F*[*V*_d_](0)} = 5*E*_c_, for each individual speckle-field instance; also, the imaginary part is fixed at Im{*F*[*V*_d_](0)} = 0. This reduces the number of active features to 40. Still, we include all *N*_*f*_ = 42 components in the feature vector, in view of future studies extended to speckle fields with varying intensities. In fact, the inactive features do not play any role in the training of the neural network.

In supervised machine learning studies it is sometimes convenient to normalize the components of the feature vector so that they have the same minimum and maximum values, or the same mean and standard deviation. This improves the efficiency in those cases in which the bare (non-normalized) feature values vary over scales that differ by several orders of magnitude. However, as can be evinced by the plot of their standard deviations (denoted *σ*[Re{*F*[*V*_*d*_](*ξ*)}] and *σ*[Im{*F*[*V*_*d*_](*ξ*)}]) in Fig. 3, the Fourier components of the speckle field differ at most by a factor of ~4. Therefore a normalization procedure is not required here.

Our plan is to train the neural network to predict the three lowest energy levels of a quantum particle in a speckle field. We generate a large number of speckle-field instances (we indicate this number with *N*_*t*_) using different random numbers, as discussed in Sec. 1. Their energy levels are computed via the finite difference approach (see Sec. 1). The target value is either the ground-state energy, or the first excited energy level, or the second energy level. Actually, for mere convenience, we consider the shifted energy levels *y* = *ε*_*i*_ = *e*_*i*_ − 〈*e*_*i*_〉_d_ (with *i* = 0, 1, 2), so that the target values have zero mean when averaged over many speckle-field instances. Each instance is represented by the feature vector ${\bf{f}}=({f}_{1},{f}_{2},\ldots ,{f}_{{N}_{f}})$, namely the *N*_*f*_ = 42 values taken from the nonzero Fourier components described above, and the target value *y* (ground state, first excited state, or second excited state) to be learned.

The statistical model we consider is a feed-forward artificial neural network, as implemented in the multi-layer perceptron regressor of the python scikit-learn library^58^. This neural network includes various layers with a specified number of neurons. The leftmost layer is the input layer. It includes *N*_*f*_ neurons, each representing one of the features values. Next, there is a tunable number of hidden layers; we indicate this number as *N*_*l*_. This is one of the details of the statistical model that will be analyzed. The number of neurons in the hidden layers, indicated as *N*_*n*_, can also be tuned. (Different hidden layers could have different numbers of neurons. However, in this article we choose to have the same number of neurons *N*_*n*_ in all hidden layers.) *N*_*n*_ is the second relevant detail of the statistical model to be analyzed. The input layer and the hidden layers also include a bias term. The rightmost layer is the output layer, and includes one neuron only. Each neuron *h* = 1, …, *N*_*n*_ in the hidden layer *l* = 1, …, *N*_*l*_ takes a value ${a}_{h}^{l}$ obtained by evaluating the so-called activation function, denoted by *g*(⋅), on the weighted sum ${\sum }_{j}\,{w}_{h,j}^{l}{a}_{j}^{l-1}$ of the values of the neurons in the previous layer *l* − 1, adding also the bias term ${b}_{h}^{l}$, leading to: ${a}_{h}^{l}=g({\sum }_{j}\,{w}_{h,j}^{l}{a}_{j}^{l-1}+{b}_{h}^{l})$. The index *j* labels neurons in the previous layer, so that *j* = 1, …, *N*_*f*_ when *l* = 1 and *j* = 1, …, *N*_*n*_ when *l* > 1. The coefficients ${w}_{h,j}^{l}$ are the weights between layer *l* and layer *l* − 1, with *l* = 1, …, *N*_*l*_ + 1. They represent the model parameters that have to be optimized during the learning process, together with the bias terms ${b}_{h}^{l}$. The neuron of the output layer (corresponding to the index *l* = *N*_*l*_ + 1) also performs the weighted sum with bias, but the activation function is here just the identity function. Taking, as an illustrative example, a neural network with one hidden layer and one neuron in the hidden layer, the learning function would be $F({\bf{f}})={w}_{1,1}^{2}\,g({\sum }_{j}\,{w}_{1,j}^{1}\,{f}_{j}+{b}_{1}^{1})+{b}_{1}^{2}$. Different choices for the activation function *g*(*x*) of the hidden neurons are possible, including, e.g., the identity, the hyperbolic tangent, and the rectified linear unit function, defined as *g*(*x*) = max(0, *x*). In this article, we adopt the latter function. A preliminary analysis has shown that other suitable choices perform quite poorly.

The training process consists in optimizing the model parameters ${w}_{h,j}^{l}$ and ${b}_{h}^{l}$ so that the function values *F*(f_t_) closely approximate the target values *y*_*t*_. Here, the index *t* = 1, …, *N*_*t*_ labels the instances in the training set. The optimization algorithm is designed to minimize the loss function $L({\bf{W}})=\frac{1}{2}{\sum }_{t}\,(F({{\bf{f}}}_{t})-{y}_{t}){)}^{2}+\frac{1}{2}\alpha {\Vert {\bf{W}}\Vert }_{2}^{2}$, where the second term is the regularization and is introduced to penalize complex models with large coefficients. It is computed with the L2-norm, indicated as ${\Vert \cdot \Vert }_{2}$, of the vector **W**, which includes all weight coefficients. The regularization is useful to avoid overfitting, the situation in which the target values of the training instances are accurately reproduced, but the neural network fails to correctly predict the target values of previously unseen instances. The magnitude of the regularization term can be tuned by varying the (positive) regularization parameter *α*. Typically, large values of *α* are required to avoid the pervasive over-fitting problem when the training set is small (if the neural network has many layers and many hidden neurons), while small (or even vanishing) values of *α* can be used if the training set is sufficiently large. The role of this parameter is another important aspect that will be analyzed below.

The optimization is performed using the Adam algorithm^59^, an improved variant of the stochastic gradient descent method, which is readily implemented in the scikit-learn library, and proves to perform better than the other available options for our problem. The tolerance parameter in the multi-layer perceptron regressor is set to 10^−10^, providing a large parameter for the maximum number of iteration so that convergence is always reached. All other parameters of the multi-layer perceptron regressor are left at their default values.

## Results

In the following, we evaluate the performance of the trained neural network in predicting the energy levels of a set of *N*_*p*_ = 40000 speckle-field instances not included in the training set. As a figure of merit, we consider the coefficient of determination, typically denoted with *R*^2^, defined in the general case as:

6 $${R}^{2}=1-\frac{\sum _{p=1}^{{N}_{p}}\,{(F({{\bf{f}}}_{p})-{y}_{p})}^{2}}{\sum _{p=1}^{{N}_{p}}\,{({y}_{p}-\bar{y})}^{2}},$$

where $\bar{y}=\frac{1}{{N}_{p}}{\sum }_{p=1}^{{N}_{p}}\,{y}_{p}$ is the average of the target values in the test set, which is essentially zero here due to the use of shifted energy levels. A perfectly accurate statistical model which exactly predicts the target values of all the instances in the test set would yield a coefficient of determination equal to *R*^2^ = 1. For example, a constant function which produces (only) the correct average of the test set target values, but (clearly) completely fails to reproduce their fluctuations, would instead correspond to the score *R*^2^ = 0. Notice that the coefficient of determination could in principle be negative in the case of an extremely inaccurate statistical model (in fact, *R*^2^ is not the square of a real number). All *R*^2^ scores reported in the following have been obtained as the average over 5 to 15 repetitions of the training of the neural network, initializing the random number generator used by the multi-layer perceptron regressor of the scikit-learn library with different seed numbers. The estimated standard deviation of the average is used to define the error bar displayed in the plots. This error bar accounts for the fluctuations due the (possibly) different local minima identified by the optimization algorithm.

**Figure 4:**
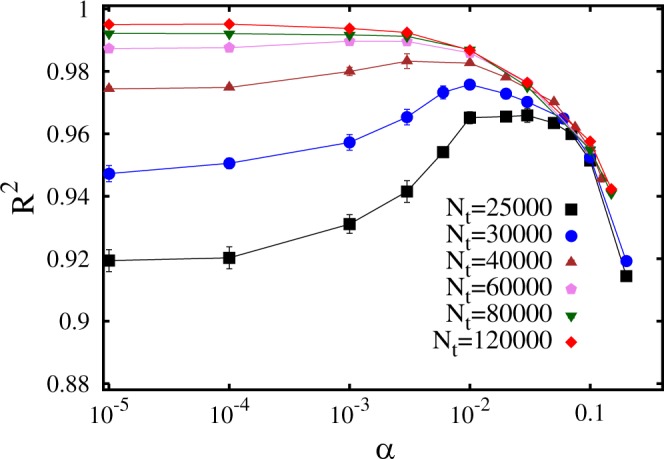
Coefficient of determination *R*^2^ as a function of the regularization parameter *α*. The different datasets correspond to different training set sizes *N*_*t*_. The neural network has N_*l*_ = 3 layers and *N*_*n*_ = 150 neurons per layer.

The first aspect of the machine learning process we analyze is the role of the regularization parameter *α*. A neural network with *N*_*l*_ = 3 hidden layers and *N*_*n*_ = 150 neurons per hidden layer is considered for this analysis, testing how accurately it predicts the (shifted) ground state energies of the *N*_*p*_ = 40000 instances of the test set. Figure 4 shows the *R*^2^ scores as a function of the regularization parameter, for different sizes of the training set *N*_*t*_. One notices that for the smallest training set with *N*_*t*_ = 25000 instances the optimal result is obtained with a significantly large regularization parameter, namely *α* ≈ 0.03. This indicates that without regularization this training set would be too small to avoid overfitting. Instead, the largest training sets provide the highest *R*^2^ scores with vanishingly small *α* values, meaning that here regularization can be avoided. In fact, this neural network proves able to accurately predict the ground-state energies of the speckle-field instances, with the highest values of the coefficient of determination *R*^2^ close to 1.

**Figure 5:**
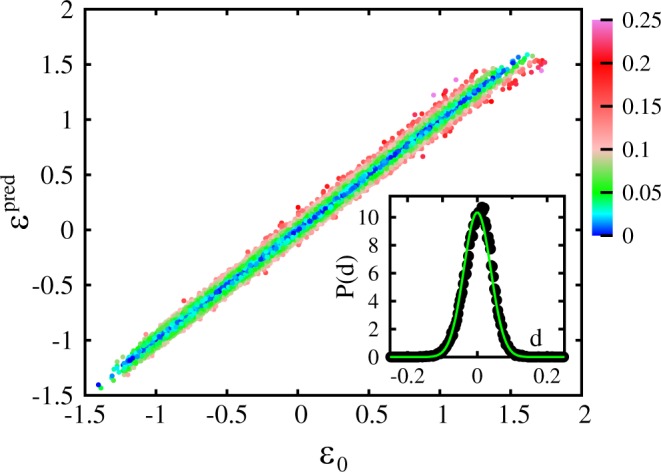
Main panel: shifted energy levels predicted by the neural network *ε*_pred_ as a function of the (shifted) exact values *ε*_0_ determined via the finite difference technique. The color scale indicates the absolute value of the discrepancy *d* = *ε*_pred_ − *ε*_0_. Inset: probability distribution of the discrepancy *d*. The (green) continuous curve is a Gaussian fitting function $P(d)=\frac{1}{\sqrt{2\pi }\sigma }\exp \,[\,\,-\,{d}^{2}\mathrm{/(2}{\sigma }^{2})]$, with the fitting parameter *σ *≅ 0.039*E*_c_.

This high accuracy can be appreciated also in the scatter plot of Fig. 5, where the shifted ground-state energy *ε*^pred^ = *F*(**f**) predicted by the neural network (with *N*_*l*_ = 3 and *N*_*n*_ = 150, as in Fig. 4) is plotted versus the exact value *ε*_0_. Here, the training set size is *N*_*t*_ = 80000, and the regularization parameter is fixed at the optimal value. The color scale indicates the absolute value of the discrepancy *d* = *ε*^pred^ − *ε*_0_. One notices that somewhat larger discrepancies occur for those speckle-field instances whose ground state energy is higher than the average. The inset of Fig. 5 displays its probability distribution *P*(*d*). This distribution turns out to be well described by a gaussian fitting function with a standard deviation as small as *σ *≅ 0.039*E*_*c*_.

**Figure 6:**
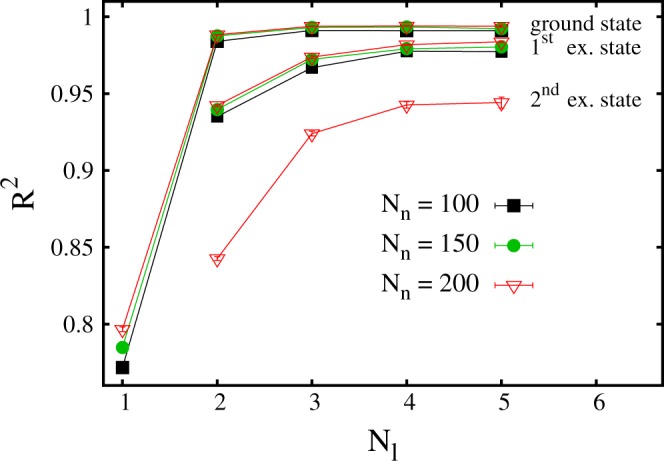
Coefficient of determination *R*^2^ as a function of the number of layers *N*_*l*_ of the neural network. The upper three datasets correspond to the ground-state energy level, for three numbers of neuron per layer *N*_*n*_. The central three datasets correspond to the first excited state. The lowest dataset corresponds to the second excited state.

**Figure 7:**
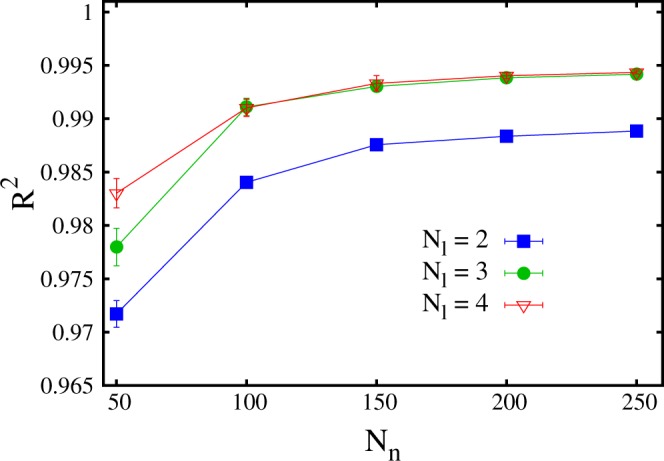
Coefficient of determination *R*^2^ for the prediction of ground state energy levels, as a function of the number of neurons per layer *N*_*n*_. The three datasets correspond to different numbers of layers *N*_*l*_.

It is interesting to analyze how the accuracy of the neural network varies with the number of hidden layers *N*_*l*_ and of the neuron per hidden layer *N*_*n*_. In Fig. 6 the *R*^2^ scores are plotted as a function of *N*_*l*_. The three upper datasets correspond to (shifted) ground-state energy predictions with three values of *N*_*n*_. In Fig. 7 the *R*^2^ scores are plotted as a function of *N*_*n*_, for three numbers of layers *N*_*l*_. The size of the training set is *N*_*t*_ = 80000. One notices that a neural network with only one hidden layer is not particularly accurate, with the *R*^2^ score being close to *R*^2^ ≈ 0.8. Instead, two hidden layers appear to be already sufficient to provide accurate predictions. Increasing the hidden layer number beyond *N*_*l*_ = 3 does not provide a sizable accuracy improvement. The number of neurons *N*_*n*_ plays a relevant role, too. A significant accuracy improvement occurs when the number of hidden neurons increases from *N*_*n*_ = 50 to *N*_*n*_ = 100. This improvement becomes less pronounced when *N*_*n*_ is increased beyond *N*_*n*_ = 150.

**Figure 8:**
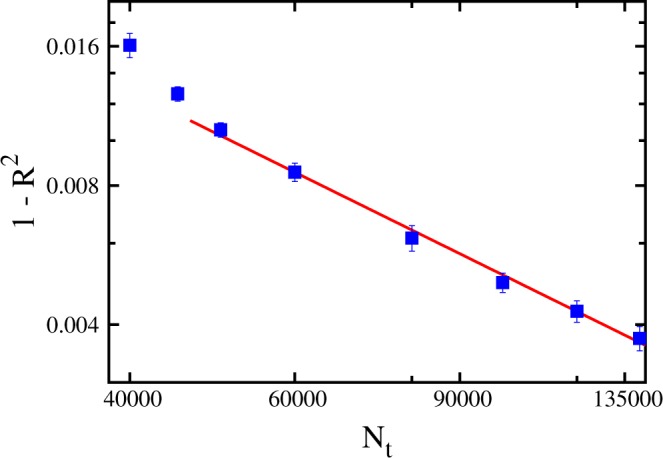
Difference between the ideal value of the coefficient of determination *R*^2^ = 1 and the actual *R*^2^-score obtained in the prediction of ground state energy levels, as a function of the training set size *N*_*t*_. The (red) line is a power-law fitting function for ${N}_{t}\ge 50000$ of the type: 1 − *R*^2^(*N*_*t*_) = *A*/*N*_*t*_, with *A* a fitting parameter. The number of layers is *N*_*l*_ = 3 and the number of neurons per layer is *N*_*n*_ = 200.

It is evident that neural networks with *N*_*l*_ > 2 and *N*_*n*_ > 150 are quite accurate statistical models to predict ground state energies; however, their *R*^2^ scores still remain close but systematically below the ideal result *R*^2^ = 1. It is possible that a larger training set would allow one to remove even this small residual error. To address this point, we plot in Fig. 8 the gap with respect to the ideal score, computed as 1 − *R*^2^, as a function of the training set size *N*_*t*_, reaching relatively large training set sizes *N*_*t*_ = 140000. The considered neural network is considerably deep and wide, having *N*_*l*_ = 3 hidden layers and *N*_*n*_ = 200 neurons per hidden layer. One observes that this gap systematically decreases with *N*_*t*_. In fact, for ${N}_{t}\ge 50000$, the gap data trend appears to be reasonably well characterized by the following power-law fitting function: 1 − *R*^2^(*N*_*t*_) = *A*/*N*_*t*_, where *A* = 513(6) is the only fitting parameter. We emphasize here that this fitting function is empirical, that it applies to the considered regime of large training set sizes *N*_*t*_, and that is is possible that the gap data would display a different scaling (e.g., logarithmically vanishing) for even larger *N*_*t*_ values. Still, the analysis of Fig. 8 suggests that, given a sufficiently large training set, a few-layers deep neural network can provide essentially arbitrarily accurate predictions of ground-state energies. Chiefly, one notices that at *N*_*t*_ = 140000 the *R*^2^ score is as accurate as *R*^2^ ≅ 0.996, meaning that the residual error is already negligible for many purposes. The training process of a deep neural network on much larger training sets becomes a computationally expensive task, at the limit of the computational resources available to us. In this regards, it is worth reminding that the computational cost of training a neural network scales with the training set size, with the number of features, and with the *N*_*l*_-th power of the number of neurons per hidden layer. In fact, in general, the inaccuracies in supervised machine learning predictions can be attributed to the limited expressive power of the statistical model (e.g., an artificial neural network with too few hidden layers), but also to the possible inability of the training algorithm to find the optimal parameters. In the case analyzed here, it appears that the training algorithm is not the limiting factor, since the *R*^2^ scores systematically improve with *N*_*t*_. However, substantial research efforts are still being devoted to improve the power and the speed of the optimization algorithms employed to train artificial neural networks, and even quantum annealing methods have recently been tested^60^.

We analyze also how accurately a neural network can predict the (shifted) excited state energies. In fact, in quantum many-body theory, predicting excited state energies is a more challenging computational problem compared to ground-state energy computations, since efficient numerical algorithms such as, e.g., quantum Monte Carlo simulations, cannot be used in general, thus demanding the use of computationally expensive techniques like exact diagonalization algorithms. It is interesting to inspect if this greater difficulty is reflected in the process of learning to predict excited state energies from previous observations. *R*^2^ scores corresponding to predictions of first excited state energies are displayed in Fig. 6, as a function of *N*_*l*_, for three numbers of neurons per hidden layer *N*_*n*_. *R*^2^ scores corresponding to the second excited state are displayed too, but for one *N*_*n*_ value only. One notices that the *R*^2^ scores are lower than in the case of ground state energy predictions, in particular the results corresponding to the second excited state. Furthermore, the number of layers appears to play a more relevant role. Deeper neural networks are necessary to get close to the ideal score *R*^2^ = 1. However, adding even more layers becomes computationally prohibitive, and is beyond the scope of this article.

It is worth mentioning that machine learning techniques have been recently employed to develop compact representations of many-body wave functions of tight-binding and quantum spin models. In particular, a specific kind of generative (shallow) neural network, namely the restricted Boltzmann machine, has been found to be capable of accurately approximating ground-state wave functions^61^. Unrestricted Boltzmann machines, including direct correlations among hidden variables, have been considered, too^62^. Interestingly, it was later found that in order to accurately represent (low-lying) excited states, deeper neural networks are required^63^, in line with our findings on energy-level approximation.

**Figure 9:**
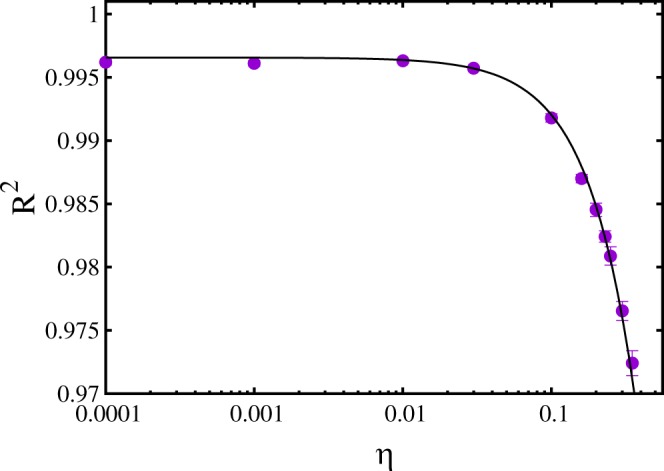
Coefficient of determination *R*^2^ for the prediction of ground state energy levels as a function of the amount of noise added to the training set. The noise is introduced by adding to the (shifted) energy levels Gaussian random variables with zero mean and standard deviation equal to the standard deviation of the energy levels of the training set times the noise-intensity parameter *η*. The (back) curve is an empirical fitting function of the type: *R*^2^(*η*) = *a* − *bη*^*c*^, with fitting parameters *a* = 0.9966(2), *b* = 0.11(1), and *c* = 1.38(7). The number of layers of the neural network is *N*_*l*_ = 3, the number of neurons per layer is *N*_*n*_ = 200, while the training set size is *N*_*t*_ = 140000.

The last aspect we investigate is the resilience of the neural network to the noise eventually present in the data representing the energy levels of the training set. Quantifying this (possible) resilience is important in order to establish if it is feasible to employ the outcomes of analog quantum simulations performed, e.g., with ultracold atom experiments, to train neural networks. In fact, while training sets obtained from numerical computations, as the exact diagonalization calculation employed here, are in general free from random fluctuations (this is not the case, e.g., of Monte Carlo simulations), in experiments a certain amount of statistical uncertainty is unavoidable. With this aim, we perform the training of a neural network on a large set of instances whose (shifted) energy levels *ε*_*i*_ have been randomly distorted by adding a Gaussian random variable with zero mean and standard deviation equal to the standard deviation of the original set of energy levels times a scale factor *η*. This scale factor quantifies the intensity of the noise added to the training set. Specifically, we consider a neural network with *N*_*l*_ = 3 layers, *N*_*n*_ = 200 neurons per layer, while the size of the training set is the largest considered in this article, namely *N*_*t*_ = 140000. The results for the coefficient of determination *R*^2^ (computed on *N*_*p*_ non-randomized test instances, previously unseen by the neural network) as a function of the noise intensity *η* are shown in Fig. 9. One notices that the prediction accuracy is essentially unaffected by the added noise up to a noise level of a few percent. Only when the noise level is above 10% (corresponding to *η* > 0.1) the reduction in the *R*^2^ score becomes significant. The *R*^2^ data appear to be well described by the empirical fitting function *R*^2^(*η*) = *a* − *bη*^*c*^, with *a*, *b*, and *c* fitting parameters. These results indicate that the neural network is capable of filtering out the signal from the noise, resulting in a remarkable resilience to the random noise present in the training set. It is worth mentioning that, analogously to the analyses reported above, the regularization parameter *α* has been optimized for each *η* value, individually. These optimizations show that, while for small added noise (small *η*) vanishingly small *α* values are optimal, meaning that no regularization is needed, larger *α* values are need when the noise intensity increases; for example, when *η* = 0.35 the optimal regularization parameter (the one providing the highest *R*^2^ score on the test set) is *α* = 0.01. This indicates that, by penalizing models with large weight coefficients, the regularization helps the neural network to avoid learning the noise, thus filtering out the signal.

These findings suggest that data obtained from cold-atom quantum simulations might be used to train neural network, possibly providing a route to make predictions on models that cannot be accurately solved via computer simulations, as for the paradigmatic case of the fermionic Hubbard model. Concerning this, it is worth mentioning that recent cold-atoms experiments implementing the fermionic Hubbard model have been analyzed via machine learning techniques^38^, and we hope that our findings will motivate further endeavours in this direction.

It is also worth mentioning that previous studies on classification problems via supervised machine learning have already found that deep neural networks are remarkably robust against noise; see, e.g., ref.^64^ and references there in. In those studies, noise was introduced in the form of many instances with random labels, even at the point of outnumbering the instances with correct labels. Our results extend these previous findings to the case of a specific, experimentally relevant, regression problem. It is also worth pointing out that techniques to reduce the effect of random errors in the training set have been developed in the machine learning community (see, e.g. ref.^65^), and that such techniques could be adapted to analyze cold-atom experiments.

## Discussion

The general problem we tried to address is whether a machine can learn to solve new quantum mechanics problems from previously solved examples. Specifically, we performed a supervised machine learning study, training a deep neural network to predict the lowest three energy levels of a quantum particle in a disordered external field. The trained neural network could be employed, e.g., to speed up the ensemble averaging calculations, for which numerous realizations have to be considered in order to faithfully represent the disorder ensemble. This kind of ensemble averaging plays a crucial role in the studies on Anderson localization (see, e.g, refs^49,50^). The quantum model we focused on is designed to describe a one-dimensional noninteracting atomic gas exposed to an optical speckle field, taking into account the structure of the spatial correlations of the local intensities of the random field. The most relevant aspects of a supervised machine learning task have been analyzed, including the number of hidden layers in the neural network, the number of neurons in each hidden layer, the size of the training set, and the magnitude of the regularization parameter. Interestingly, we found that a neural network with three or four layers of hidden neurons can provide extremely accurate prediction of ground-state energies using for training a computationally feasible number of speckle-field instances. The predictions of excited state energies turned out to be slightly less accurate, requiring deeper neural networks to approach the optimal result. We also quantified the amount of regularization required to avoid the overfitting problem in the case of small or moderately large training sets.

In recent years, the experiments performed with ultracold atoms have emerged as an ideal platform to perform quantum simulations of complex quantum phenomena observed also in other, less accessible and less tunable, condensed matter systems. In the long term, one can envision the use of cold-atom setups to train artificial neural network to solve problems that challenge many-body theorists, like the many-fermion problem. In the medium term, these experiments can be employed as a testbed to develop efficient representations of instances of quantum systems for supervised machine learning tasks, as well as for testing the accuracy of different statistical models, including, e.g, artificial neural networks, convolutional neural networks, gaussian approximation potentials, or support vector machines^19,24^. These machine learning techniques could find use in particular in the determination of potential energy surfaces for electronic structure simulations^29^, or even in ligand-protein affinity calculations for drug-design research. For this purpose, it is of outmost importance to understand how accurate the above mentioned statistical models can be in predicting the energy levels of complex quantum many body systems. This is one of the reasons that motivated our study. In view of the possible future use of cold-atom quantum simulators as computational engines to provide training sets for supervised machine learning tasks, we investigated the resilience of artificial neural networks to noise in the training data, since this is always present is any experimental result. We found that a deep neural network with three layers is remarkably robust to such noise, even up to a 10% noise level in the target values of the training data. This level of accuracy is indeed within the reach of cold-atoms experiments. This is an important result suggesting that training artificial neural networks using data obtained from cold-atom quantum simulations would indeed be feasible. The analysis on the amount of regularization discussed above provides information on how many experimental measurements would be needed to avoid the risk of overfitting.

It well known that an accurate selection of the features used to represent the system instances can greatly enhance the power of supervised machine learning approaches. In this article we have employed the Fourier components of the optical speckle field. This appears to be an effective choice for systems characterized by external fields with spatial correlations. This approach could be further improved by combining this choice of features with different types of artificial neural networks as, e.g., the convolutional neural networks; the latter have in fact been considered in ref.^19^, but in combination with a real-space representation of the quantum system. In this regards, it is worth mentioning that various alternative representations have been considered in the field of atomistic simulations for many-particle systems, including, e.g, the atom-centered symmetry functions^26^, the neighbor density, the smooth overlap of atomic positions, the Coulomb matrices (see, e.g, ref.^29^), and the bag of bonds model^13^. In this context, an important open problem is the development of space-scalable representations, and associated statistical models, that can be applied to systems of increasing system size. Previous machine-learning studies on atomistic systems exploited the locality of atomic interaction to build such scalable models for many-atom systems^22,29^. This property, which is sometimes referred to as nearsightedness, characterizes many common chemical systems. However, quantum mechanical systems often host long-range correlations that cannot be captured by locality-based models. A more general approach, which will be the focus of future investigations, might be built using transfer-learning techniques^66^ whereby models optimized on small scale systems form the building blocks of neural-network models for large scale systems with a moderate computational cost.

## Data Availability

All data sets and computer codes employed in this work are available upon request.
